# A case of rapid and extensive cardiac myocyte microcalcification in a patient admitted to intensive care following out-of-hospital cardiac arrest

**DOI:** 10.1186/s42047-026-00232-9

**Published:** 2026-04-20

**Authors:** Gayath Bimal Jayarathna, Joseph Westaby, Mary N. Sheppard

**Affiliations:** 1 Postgraduate Institute of Medicine, University of Colombo, Colombo, Sri Lanka; 2 CRY Cardiovascular Pathology Unit, St George’s, Cardiovascular and Genetic Research Institute, University of London, London, SW17 0RE UK

**Keywords:** Intramyocyte microcalcification, Out-of-hospital cardiac arrest, Subendocardial haemorrhagic infarction

## Abstract

**Background:**

Early myocardial calcification is an uncommon histopathological finding and is usually associated with chronic myocardial injury. Rapid intramyocyte microcalcification following global ischemia is rarely described.

**Case presentation:**

A 42-year-old previously healthy male suffered an out-of-hospital cardiac arrest while cycling. Following prolonged cardiopulmonary resuscitation and return of spontaneous circulation, he was admitted to ITU before being confirmed to have Hypoxic Ischemic Encephalopathy not compatible with life. Despite intensive supportive management for multiorgan failure patient was pronounced dead within 48 h of admission. There was no family history of heart disease or sudden cardiac death. The comprehensive forensic autopsy examination revealed no significant traumatic injuries that could account for death. Specialized cardiac examination showed subendocardial haemorrhagic infarction over the left ventricle. Histological examination demonstrated extensive focal intramyocyte microcalcification affecting sub endocardium of both ventricles in the areas of infarction with absence of fibrosis or inflammation.

**Conclusion:**

This case highlights rapid dystrophic intramyocyte microcalcification as a rare histological manifestation of severe global myocardial ischemia following cardiac arrest and resuscitation. Autopsy pathologists should be aware that subendocardial infarction with or without intramyocyte microcalcification and HIE occur secondary to cardiac arrest in those admitted to ITU and it is essential to investigate the cause of the primary cardiac arrest.

## Introduction

Out-of-Hospital Cardiac Arrest (OHCA) remains a leading cause of global mortality, accounting for millions of deaths worldwide each year (Myat et al. 2018). Despite advances in emergency medical care, overall survival rates remain low, with pooled survival estimates ranging from 7.6% to 8.8% (Fothergill et al. 2013). Survival depends on early initiation of basic life support, timely advanced life support (ALS), and optimal post-resuscitation care (Deakin 2018). OHCA with prolonged downtime frequently results in severe hypoxic-ischemic brain injury (HIBI), which is a major contributor for mortality among patients who achieve initial return of spontaneous circulation (ROSC) (Sheppard 2015, Singer et al. 2024). Prolonged cerebral hypoxia and hypoperfusion result in irreversible neuronal injury and secondary pathological processes, culminating in coma, a persistent vegetative state, or brain death. We report a case of OHCA with prolonged resuscitation and return of spontaneous circulation, followed by death in 48 h, in which cardiac histopathology revealed rapid and extensive intramyocyte microcalcification.

## Case presentation

A 42-year-old previously healthy male collapsed while cycling, an event witnessed by a companion. Immediate bystander cardiopulmonary resuscitation (CPR) was initiated and subsequently continued by emergency medical services using ALS protocols. The initial cardiac rhythm was pulseless electrical activity, which later deteriorated into ventricular fibrillation. A total of six cycles of CPR were administered, and the patient was endotracheally intubated at the scene. ROSC was achieved after approximately 60 min of resuscitative efforts.

Computed tomography pulmonary angiography performed on admission showed no evidence of traumatic visceral injury or pulmonary embolism. Computed tomography of the brain demonstrated features consistent with HIBI. Bedside echocardiography revealed global hypokinesia with a reduced ejection fraction of approximately 40% and no valvular abnormalities. Coronary angiography demonstrated normal coronary arteries. Despite intensive supportive management for multiorgan failure in the intensive care unit patient was pronounced dead within 48 h of admission.

### Post-mortem and specialized cardiac examination

A comprehensive autopsy examination was performed by the local hospital forensic pathologist. There was no family history of heart disease or sudden cardiac death. There were no reported symptoms such as chest pain, palpitations or syncope. He had no significant past medical history, especially hypertension or renal disease. External examination revealed multiple superficial healing abrasions predominantly over bony prominences, consistent with a fall from a bicycle, without any significant traumatic injuries that could account for death. The autopsy demonstrated moderate cerebral oedema, ventilated lungs, and numerous petechial haemorrhages over the pericardial surface of the heart in keeping with resuscitation and admission to ITU. There was no evidence of renal cortical scarring suggestive of hypertension. Toxicological analysis was negative and did not identify any drugs at levels associated with toxicity or death. Histopathological examination of the vital organs, with the exception of the heart, was unremarkable. The undissected heart was referred to the CRY Cardiovascular Pathology Unit at St George’s, University of London, for specialized cardiac examination (Westaby et al. 2022). 

Routine cardiac dissection was performed and measurements of the cardiac chambers, including ventricular wall thickness and chamber diameters, were obtained. A standard sampling protocol was followed, collecting 7 samples representing right and left coronary arteries, right ventricular outflow tract, septum and anterior, lateral and inferior right and left ventricles. All the sections were stained with Hematoxylin and Eosin (H&E) staining. Only in cases with heart block additional samples are obtained of to access the SA, AV nodes and conduction pathways. In this case no conduction system analysis or molecular autopsy were performed.

**Fig. 1:**
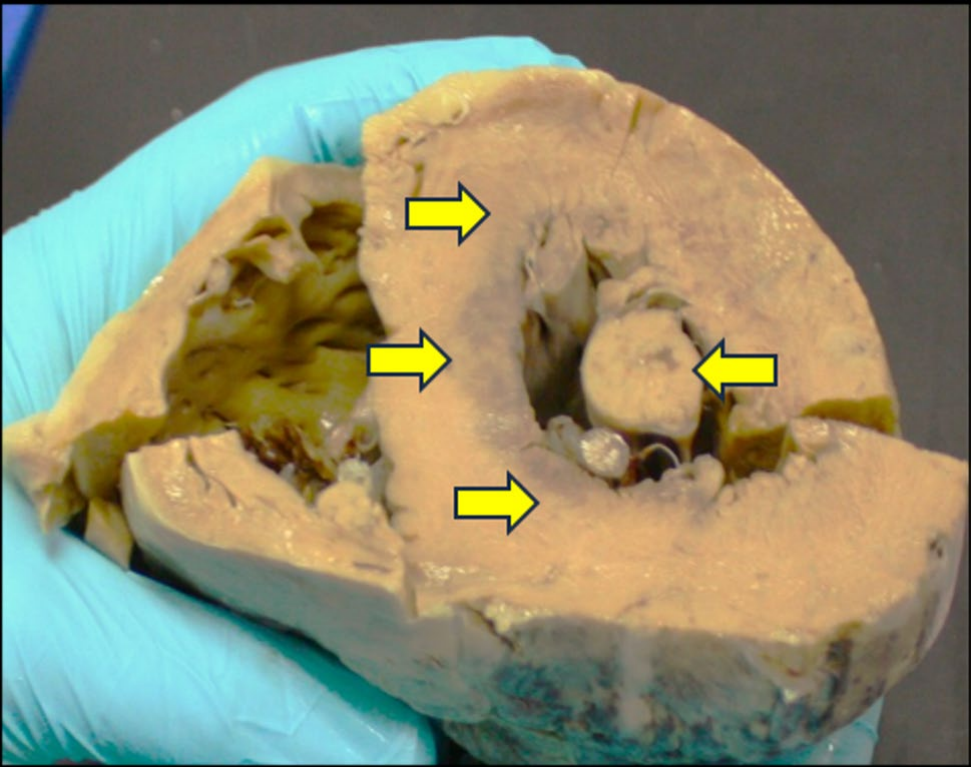
Cross section across the ventricles and septum showing subendocardial haemorrhagic infarction (yellow arrows) over the septum, anterior and posterior walls of the left ventricle as well as over the papillary muscle

**Fig. 2:**
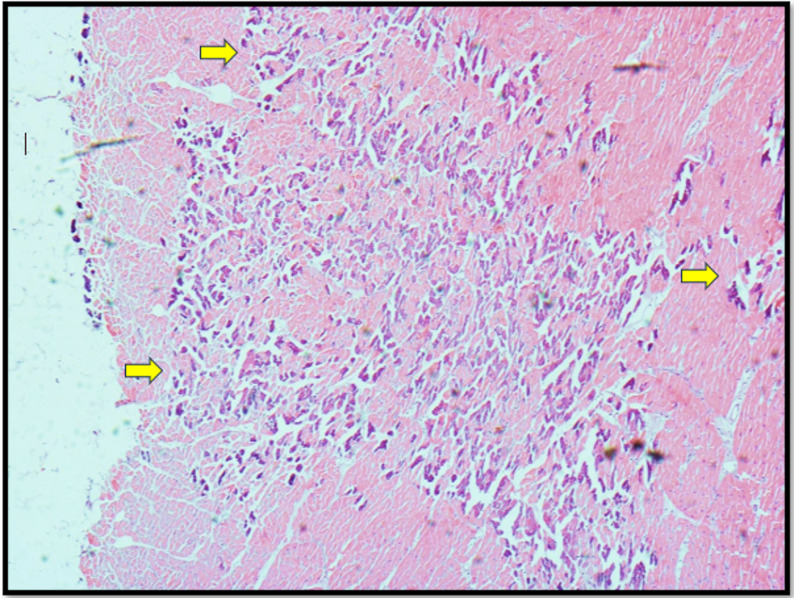
Histological section of left ventricular myocardium (H & E × 10) demonstrating focal subendocardial myocardial necrosis with myocyte calcification (yellow arrows) without interstitial fibrosis

**Fig. 3:**
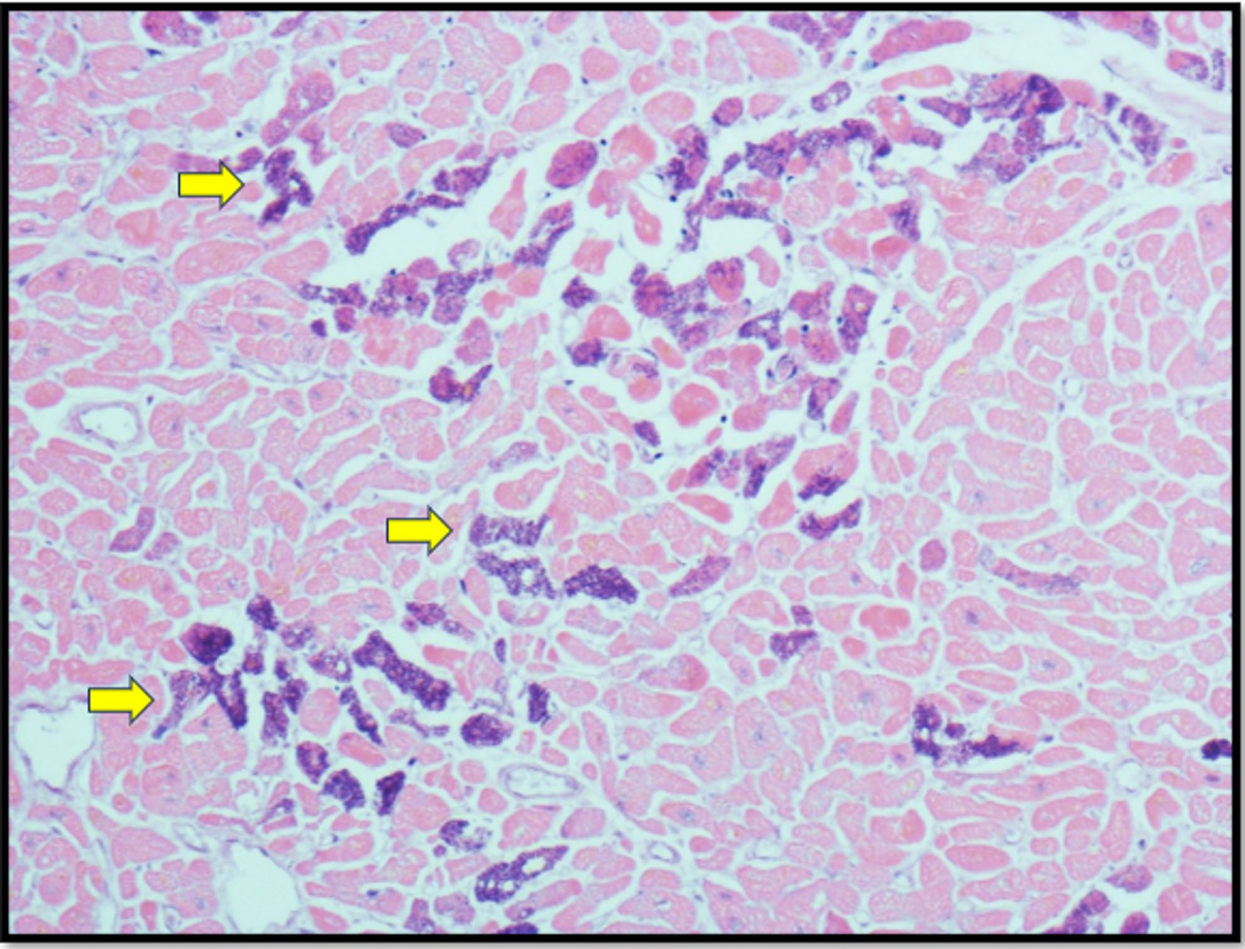
Histological section of left ventricular myocardium (H & E × 40) demonstrating intramyocyte microcalcification (Yellow arrows) in the absence of inflammatory infiltrates

The heart weighed 483 g and it was mildly enlarged. No macroscopic evidence of significant coronary artery disease, chamber dilatation, ventricular hypertrophy, valvular abnormality, or fatty infiltration was observed. There was subendocardial haemorrhagic infarction over the septum, anterior and inferior walls of the left ventricle as well as over the papillary muscle (Fig. 1) which can be a common finding with survival after cardiac arrest. Histological examination confirmed multiple areas of subendocardial myocyte necrosis (Fig. 2) with significant microcalcifications within the sarcoplasm of myocytes (Fig. 3) in both the right and left ventricles. These myocyte microcalcifications was only demonstrated in the areas of myocyte necrosis. The Myocardial architecture was preserved without interstitial or replacement fibrosis and inflammation. No associated inflammatory cell infiltrates were identified around the necrotic myocytes. The heart was otherwise histologically normal. The heart was otherwise histologically normal.

## Discussion

The presence of myocardial microcalcification following cardiac arrest is a specific pathological hallmark of irreversible cell death and subsequent mineral dysregulation. Unlike metastatic calcification (which is caused by elevated serum calcium levels), dystrophic calcification occurs in dying or necrotic tissue despite normal systemic calcium concentrations. Cardiac arrest leads to immediate ATP depletion. In the absence of ATP, the sarcolemmal Ca²⁺-ATPase and Na⁺/K⁺-ATPase pumps fail. Consequently, extracellular calcium rapidly enters the cytosol. Because the sarcoplasmic reticulum can no longer effectively sequester calcium, the intracellular concentration rises markedly. The mitochondria attempt to buffer this excess calcium; however, at high concentrations, calcium reacts with phosphate to form hydroxyapatite crystals within the mitochondrial matrix (Nance et al. 2015). 

These microscopic deposits appear as granular, basophilic (blue-purple) inclusions on H&E staining within the cytoplasm of necrotic myocytes. When blood flow is restored with ROSC, a substantial influx of extracellular calcium is delivered to cells with already compromised membranes of the necrotic cells, providing a substrate for rapid mineralization. In addition, reperfusion generates reactive oxygen species (ROS), which cause further lipid bilayer damage and increase cellular permeability to calcium. If microcalcifications are widespread and prominent on histological examination, this strongly suggests a survival interval of at least 24–72 h following cardiac arrest (Buschmann et al. 2013). Therefore, the extensive microcalcification in the index case is strongly consistent with the reported survival interval of 48 h.

Recent reports, Kido et al., a Japanese systematic review, have highlighted rapid myocardial calcification across diverse pathological settings (Kido et al. 2024). Experimental animal models suggest that ischemia-induced disruption of intracellular calcium homeostasis plays a central role. Mitochondrial dysfunction, sarcolemma membrane damage, and impaired calcium sequestration result in calcium influx into injured myocytes, culminating in calcium phosphate deposition within mitochondria and the sarcoplasm (Wu et al. 2001, Tavernier et al. 2001, Santulli et al. 2015, Nicolo et al. 2023). Topaz and Lili J et al., reports rapid myocardial calcification has been described in paediatric patients with congenital heart disease and in adults with metabolic disturbances such as chronic renal failure (Topaz 1986, Li et al. 2021). In most cases, myocardial calcification is detected incidentally on computed tomography and is associated with poor prognosis. In the present case, prolonged global ischemia during an estimated 60-minute downtime, followed by short-term survival (48 h) after resuscitation, likely contributed to the rapid development of intramyocyte microcalcification.

The pathology of subendocardial infarction following resuscitation and survival after out-of-hospital cardiac arrest has been described in previous studies and may lead to misinterpretation as primary myocardial pathology, with potential implications for the diagnosis of sudden arrhythmic death syndrome (Coelho-Lima et al. 2022, Sheppard et al. 2023). The calcification represents a secondary manifestation of severe ischemic myocardial injury following cardiac arrest. The patient had already sustained irreversible HIBI at the time of admission. Although intensive care support maintained somatic organ failure, neurological viability had been irreversibly lost as a consequence of prolonged cerebral hypoxia during cardiac arrest. Therefore, the immediate pathological cause of death in this case was sudden adult death with a morphologically normal heart, which is a recognized as sudden adult death syndrome (SADS) in a state of possible electrical abnormalities (Najim Lahrouchi et al. 2017). Family screening for probable genetic mutation in channelopathy genes would be helpful in this case while the cause of the initial cardiac arrest remains undetermined.

## Conclusion

This case demonstrates that cardiac myocyte microcalcification can develop rapidly and extensively, with a predominantly subendocardial distribution, following prolonged out-of-hospital cardiac arrest and resuscitation. In individuals admitted to the intensive care unit after ROSC, subendocardial infarction is a frequent secondary finding, reflecting global myocardial hypoperfusion rather than a primary lethal cardiac pathology. The subsequent histopathological myocardial changes therefore represent post-resuscitative and hypoxic injury, rather than the initiating event leading to death.

## Data Availability

No datasets were generated or analysed during the current study.
